# Continuous control of tracheal cuff pressure for VAP prevention: a collaborative meta-analysis of individual participant data

**DOI:** 10.1186/s13613-015-0087-3

**Published:** 2015-11-24

**Authors:** Saad Nseir, Leonardo Lorente, Miquel Ferrer, Anahita Rouzé, Oswaldo Gonzalez, Gianluigi Li Bassi, Alain Duhamel, Antoni Torres

**Affiliations:** CHU Lille, Centre de Réanimation, 59000 Lille, France; Faculté de Médecine, Université Lille, 59000 Lille, France; Intensive Care Unit, Hospital Universitario de Canarias, Tenerife, La Laguna, Spain; Respiratory Intensive and Intermediate Care Unit, Department of Pneumology, Hospital Clinic, Institute of Thorax, Barcelona, Spain; Epidemiology, Public Health and Quality of Care, Nord-de-France University, Lille, France

**Keywords:** Cuff pressure, Pneumonia, Mechanical ventilation, Microaspiration, Critical care, Meta-analysis

## Abstract

**Background:**

Underinflation of tracheal cuff is a risk factor for microaspiration of contaminated secretions and subsequent ventilator-associated pneumonia (VAP). The aim of this collaborative meta-analysis of individual participant data is to determine the impact of continuous control of *P*_cuff_ on the incidence of VAP.

**Methods:**

Studies were identified by searching PubMed and references of relevant articles. Data from 3 prospective controlled trials (two randomized and one quasi-randomized), which evaluated the impact of continuous control of *P*_cuff_ on the incidence of VAP, were obtained and pooled together. Three different devices were used to continuously control *P*_cuff_. VAP was diagnosed using clinical, radiologic, and quantitative microbiological criteria. The impact of continuous control of *P*_cuff_ on VAP was assessed by Cox regression analysis, stratified on trial.

**Results:**

263 (48.4 %) patients received continuous control of *P*_cuff_, and 280 (51.5 %) patients received routine control of *P*_cuff_ using a manometer. 36 (13.6 %) VAP were diagnosed in continuous control group, and 72 (25.7 %) in routine care group (HR 0.47, 95 % CI 0.31–0.71, *p* < 0.001). However, heterogeneity was apparent in continuous control effect size across trials (*I*^2^ = 58 %, *p* = 0.085). The number of patients needed to treat to prevent one VAP episode was 8. No significant impact of continuous control of *P*_cuff_ was found on duration of mechanical ventilation, ICU length of stay, or mortality.

**Conclusion:**

Continuous control of *P*_cuff_ might be beneficial in reducing the risk for VAP. However, no significant impact of continuous control of *P*_cuff_ was found on duration of mechanical ventilation, ICU length of stay, or mortality.

## Background

Prevention of ventilator-associated pneumonia (VAP) is an important strategy to improve the quality of care provided to critically ill patients [1–3]. While VAP-attributable mortality is still a matter for debate [4], this ICU-acquired infection is associated with increased antimicrobial use and duration of mechanical ventilation [5, 6]. Important progress has been achieved during the last two decades in the understanding of pathophysiology of VAP, resulting in improvement in prevention strategies and reduced incidence of VAP [7, 8].

Microaspiration of contaminated oropharyngeal and gastric secretions is the main mechanism of entry of bacteria into the lower respiratory tract [9, 10]. Local and general host defenses play an important role in the progression from colonization to VAP [11]. The quantity and virulence of bacteria are also important factors in this process [12]. Several measures have been studied for prevention of microaspiration in intubated critically ill patient. These could be classified into enteral nutrition, mechanical ventilation, tracheal tube, and patient-related factors [13]. With regards to tracheal tube, several recent studies investigated how sealing could be improved, in order to avoid microaspiration. Subglottic secretion drainage has been shown to significantly reduce VAP incidence, antimicrobial use, and duration of mechanical ventilation [14, 15]. Other preventive measures related to tracheal tube, such as polyurethane cuff and conical cuff shape have been suggested. However, a recent randomized controlled multicenter study did not report any significant impact of these measures regarding the rate of tracheobronchial colonization, or VAP [16].

Underinflation of tracheal cuff (<20 cmH_2_O) is a well-known risk factor for microaspiration and VAP [17]. Therefore, it is recommended to adjust cuff pressure (*P*_cuff_) around 25 cmH_2_O using a manometer, to prevent complications related to underinflation and overinflation of tracheal cuff [18]. However, in spite of routine control of *P*_cuff_ using a manometer, underinflation and overinflation are very common in intubated patients [19, 20]. Continuous control of *P*_cuff_ has been suggested to improve tracheal sealing and to prevent VAP. Three prospective trials evaluated the impact of continuous control of *P*_cuff_ on the incidence of VAP [21–23]. However, all these studies were performed in single centers and reported different results. Therefore, we performed this collaborative-pooled meta-analysis to determine the impact of continuous control of *P*_cuff_ on the incidence of VAP in critically ill adult patients.

## Methods

We established a collaboration to undertake this meta-analysis of individual patient data. We included all prospective trials, which evaluated the impact of continuous control of *P*_cuff_ on the incidence of VAP.

### Ethical aspects

The three studies used for this pooled analysis have been approved by local institutional regulatory boards. Informed consent was obtained from all patients, or from their next of kin.

### Search for eligible trials

We identified clinical prospective clinical trials that compared the continuous control of *P*_cuff_ and routine care regarding the incidence of VAP. We searched PubMed (from January 1995 through June 2015), using the terms “continuous control of tracheal cuff pressure,” “continuous control of endotracheal cuff pressure,” and the term “ventilator-associated pneumonia.” We also searched references of relevant articles. Studies comparing continuous control of *P*_cuff_ and another intervention versus routine care were excluded, because it is impossible to determine the exact impact of continuous control of *P*_cuff_ on VAP rate in these studies.

### Outcomes

The primary outcome was the incidence of VAP. Secondary outcomes included duration of mechanical ventilation, mechanical ventilation-free days, duration of antimicrobial treatment, length of ICU stay, and ICU mortality.

### Collected data

The rationale for choice of factors was based on prior association with outcome. At ICU admission: age, acute physiology and chronic health evaluation (APACHE) II score, sequential organ failure assessment (SOFA) score, male gender, cause for ICU admission, type of admission, diabetes mellitus, chronic obstructive pulmonary disease (COPD), chronic heart failure, cirrhosis, chronic renal failure, and immunosuppression. During ICU stay: SOFA score at randomization, subglottic secretion drainage, duration of mechanical ventilation before randomization, sucralfate, proton-pump inhibitor, or H2 receptor antagonist use, reintubation, mean *P*_cuff_, percentage of *P*_cuff_ measurements <20 cmH_2_O, underinflation of *P*_cuff_ (<20 cmH_2_O), overinflation of *P*_cuff_ (>30 cmH_2_O), mean positive end expiratory pressure (PEEP) level, sedation, Ramsay score, head of bed elevation, paralytic agent use, red blood cell transfusion, enteral nutrition, ventilator-associated tracheobronchitis (VAT) [24], and tracheostomy. At VAP diagnosis: polymicrobial VAP and type of microorganisms.

### VAP definition

VAP was defined as the presence of new or progressive pulmonary infiltrate and at least two of the following criteria: (a) fever (≥38 °C) or hypothermia (<36 °C), (b) leukocytosis (>12 × 10^9^/L) or leukopenia (<3.5 × 10^9^/L), and (c) purulent respiratory secretions. Microbiological confirmation was required in all patients (positive bronchoalveolar lavage ≥10^4^ cfu/mL, or positive tracheal aspirate ≥10^5^ cfu/mL) [25]. Only first episodes of VAP diagnosed > 48 h after starting mechanical ventilation were taken into account.

### Statistical analysis

Quantitative variables are expressed as mean [±standard deviation (SD)] in case of Gaussian distribution, or median [interquartile range (IQR)] otherwise. Normality was examined using histograms and Shapiro–Wilk test. Qualitative variables are expressed as numbers (percentage). Patient characteristics at ICU admission and during ICU stay; and secondary outcomes were compared between the two study groups using Student t-test for quantitative variables (Mann–Whitney *U* test was used for non-Gaussian distribution) and Chi-square test for qualitative variables (Fisher’s exact was used when the expected cell frequency was <5).

The probability of VAP occurrence over time was compared between the two study group using a Cox proportional hazard model stratified on trial. Heterogeneity across trials was examined by formal interaction test and quantified by calculating the *I*^2^. Patients were censored at the time of death or extubation. We performed an exclusion sensitivity analysis to evaluate the contribution of individual studies to the overall pooled estimate. A sensitivity analysis excluding patients receiving subglottic secretion drainage was also performed.

In both groups pooled together, we used a Cox proportional hazard model stratified on trial to identify factors associated with the occurrence of VAP. All variables with a *p* value < 0.20 were included into a backward-stepwise Cox regression analysis.

Statistical analysis was done at the 2-tailed α level of 0.05, except tests for the homogeneity in which an α level of 0.10 was chosen. Data were analyzed with the SAS software package, version 9.3 (SAS Institute, Cary, NC, USA).

## Results

### Study characteristics

**Fig. 1:**
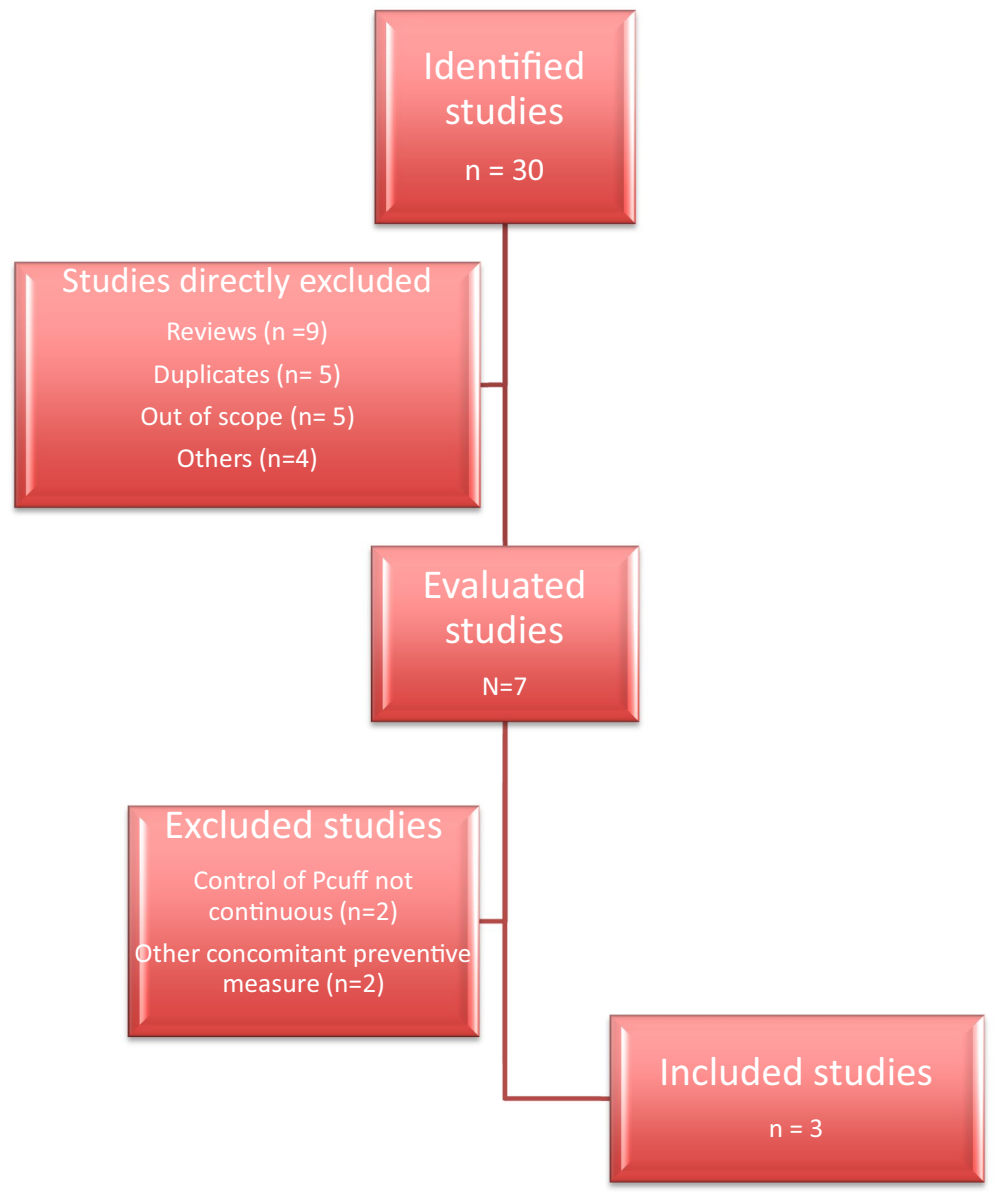
Studies evaluated for inclusion in this analysis

Among the 30 identified studies, 23 studies were directly excluded (reviews 9, duplicates 5, out of scope 5, others 4). Among the 7 remaining studies, 2 were excluded because *P*_cuff_ control was not continuous, and 2 because other concomitant preventive measures were used in the intervention group (Fig. 1).

Data from 543 patients were obtained from the three included trials, representing 99 % of all included patients (5 patients were excluded for missing data). Two studies were randomized controlled and one was quasi-randomized controlled (patients who were admitted to an odd-numbered ICU cubicle received continuous control of *P*_cuff_, and those admitted to an even-numbered ICU cubicle received routine care). Three different devices (Mallinckrodt Pressure Control^®^, VBM Medizintechnik GmbH, Sulz am Neckar, Germany; Nosten^®^, Leved, Saint Maur, France; and an electronic artisanal device) were used to continuously control *P*_cuff_. Subglottic secretion drainage was used in some patients included in one trial. All tracheal tubes were polyvinyl chloride (PVC), standard shape—cuffed. Characteristics of the three studies are presented in Table 1.

**Table 1:** Characteristics of studies assessing the impact of continuous control of cuff pressure on the incidence of ventilator-associated pneumonia

	Valencia et al. [21]	Nseir et al. [22]	Lorente et al. [23]
Number of included patients	137	122	284
Type of study	Randomized controlled	Randomized controlled	Quasi-randomized controlled
Primary objective	VAP	Microaspiration	VAP
Device	Electronic	Pneumatic	Electronic
Target *P*_cuff_ (cmH_2_O)	25	25	25
Surgical patients	28	0	28
Chronic respiratory disorders	38	27	15
VAP preventive measures			
Oral care	CHX 0.12 % X3/days	CHX 0.10 % X3/days	CHX 0.12 % X3/days
Semirecumbent position	Yes	Yes	Yes
Subglottic secretion drainage	No	No	Yes
VAP incidence in control group	15	26	22
Reduction in VAP rate	NS	62	51

Results are %, unless otherwise specified

*VAP* ventilator-associated pneumonia, *CHX* chlorhexidine, *NS* not significant

### Patient characteristics

Two hundred and sixty-three (48 %) patients received continuous control of *P*_cuff_ and 280 (52 %) received routine care using a manometer. No significant difference was found in patient characteristics at ICU admission between patients who received continuous control of *P*_cuff_, and those who received routine care (Table 2). While mean *P*_cuff_ was significantly higher in patients who received continuous control of *P*_cuff_ compared with those who received routine care, rate of patients with underinflation of *P*_cuff_, with overinflation of *P*_cuff_, and percentage of *P*_cuff_ measurements <20 cmH_2_O were significantly lower in patients with continuous control of *P*_cuff_ compared with those who received routine care. Other patient characteristics during ICU stay were similar in the two groups (Table 3).

**Table 2:** Patient characteristics at ICU admission

	Continuous control of *P*_cuff_		
	Yes (*n* = 263)	No (*n* = 280)	*p* value
Age, years, mean ± SD	61 ± 16	63 ± 15	0.141
APACHE II score	18 (13, 23)	18 (13, 23)	0.624
SOFA score	5 (3, 7)	5 (3, 8)	0.424
Male gender	177 (67)	172 (61)	0.181
Direct admission	86 (33)	86 (31)	>0.999
Cause for admission^a^			
Cardiac surgery	23 (10)	26 (9)	0.944
Cardiovascular failure	52 (20)	49 (17)	0.569
Respiratory failure	98 (37)	104 (37)	>0.999
Digestive failure	25 (9)	27 (10)	>0.999
Neurologic failure	48 (18)	52 (18)	>0.999
Others	18 (7)	24 (8)	0.554
Type of admission			0.868
Surgical	57 (22)	62 (22)	
Medical	182 (69)	196 (70)	
Trauma	24 (9)	22 (8)	
Diabetes mellitus	57 (22)	71 (25)	0.363
COPD	65 (25)	66 (23)	0.833
Chronic heart failure	48 (18)	43 (15)	0.431
Cirrhosis	22 (8)	16 (6)	0.298
Chronic renal failure	16 (6)	23 (8)	0.427
Immunosuppression	39 (15)	44 (16)	0.867
Study			0.828
1	68 (26)	69 (25)	
2	61 (23)	61 (22)	
3	134 (51)	150 (53)	

Data are number (%), or median (IQR); unless otherwise specified

*APACHE* acute physiology and chronic health evaluation, *SOFA* sequential organ failure assessment

^a^Some patients had more than one cause for ICU admission

**Table 3:** Patient characteristics during ICU stay

	**Continuous control of** *P*_cuff_		
	Yes (*n* = 263)	No (*n* = 280)	*p* value
SOFA score at randomization	4 (1, 7)	4 (2, 6)	0.538
Subglottic secretion drainage	53 (20)	65 (23)	0.447
Antimicrobial treatment	237 (90)	260 (93)	0.321
MV duration before randomization	0 (0, 1)	0 (0, 1)	0.531
Sucralfate	42 (16)	45 (16)	>0.999
Proton-pump inhibitor	182 (69)	180 (64)	0.732
H2 receptor antagonists	31 (12)	47 (17)	0.124
Reintubation	41 (15)	30 (11)	0.120
Mean *P*_cuff_	25 (24, 26)	22 (21, 24)	<0.001
Underinflation of *P*_cuff_	2 (1)	118 (42)	<0.001
% *P*_cuff_ measurements <20 cmH_2_O	0 (0,0)	16 (0, 18)	<0.001
Overinflation of *P*_cuff_	8 (3)	82 (29)	<0.001
Mean PEEP (cmH_2_O)	5 (5, 5)	5 (5, 5)	0.358
Sedation	235 (89)	253 (90)	0.806
Ramsay score	4 (3, 4)	4 (3, 4)	0.432
HOB elevation (°)	37 (30, 40)	35 (30, 40)	0.508
Paralytic agent use	22 (8)	32 (11)	0.315
Red blood cell transfusion	77 (29)	73 (26)	0.359
Enteral nutrition	178 (68)	195 (70)	0.689
Tracheostomy	49 (19)	41 (15)	0.257

Data are number (%), or median (IQR)

*SOFA* sequential organ failure assessment, *MV* mechanical ventilation, *P*_*cuff*_ cuff pressure, *PEEP* positive end expiratory pressure, *HOB* head of bed

### Characteristics of VAP patients

One hundred and eight (19.8 %) patients developed at least one VAP episode. Early-onset and late-onset pneumonia represented 49 and 51 % of VAP episodes, respectively. Duration of mechanical ventilation [5 (2, 10) vs 6 (3, 10) days, *p* = 0.323] and percentage of patients with early-onset or late-onset VAP (8 vs 12 %, 6 vs 14 %, *p* = 0.153; respectively) were similar in patients with continuous control of *P*_cuff_ compared with patients with routine care. Ten patients (9 %) had polymicrobial VAP. Gram-negative bacteria and MDR bacteria represented 84 and 36 % of microorganisms responsible for VAP episodes, respectively. *Pseudomonas aeruginosa* was the most frequently identified microorganism (16 %), followed by *Staphylococcus aureus* (10 %), and *Enterobacter* spp. (10 %) (Table 4).

**Table 4:** Microorganisms responsible for ventilator-associated pneumonia

	Continuous control of *P*_cuff_	
	Yes (*n* = 36)	No (*n* = 72)
Microorganisms (*n*)	38	80
Polymicrobial VAP	2 (5)	8 (11)
MDR bacteria	13 (36)	30 (42)
Gram-negative	33 (92)	66 (92)
*Pseudomonas aeruginosa*	5 (14)	14 (19)
Enterobacter species	3 (8)	9 (12)
*Escherichia coli*	6 (17)	5 (7)
*Citrobacter freundii*	1 (3)	4 (5)
*Acinetobacter baumannii*	2 (5)	5 (7)
*Haemophilus influenzae*	8 (22)	8 (11)
*Stenotrophomonas maltophilia*	1 (3)	4 (5)
*Klebsiella oxytoca*	2 (5)	6 (8)
Serratia species	2 (5)	7 (10)
Others	3 (8)	4 (5)
Gram-positive	5 (14)	14 (19)
Methicillin-resistant *S. aureus*	1 (3)	7 (10)
Methicillin-sensitive *S. aureus*	1 (3)	3 (4)
*Streptococcus pneumoniae*	1 (3)	2 (3)
Others	2 (5)	2 (3)

*VAP* ventilator-associated pneumonia, *MDR* multidrug resistant

Data are number (%)

*p* > 0.2 for all comparisons

### Impact of continuous control of *P*_cuff_ on outcomes

**Fig. 2:**
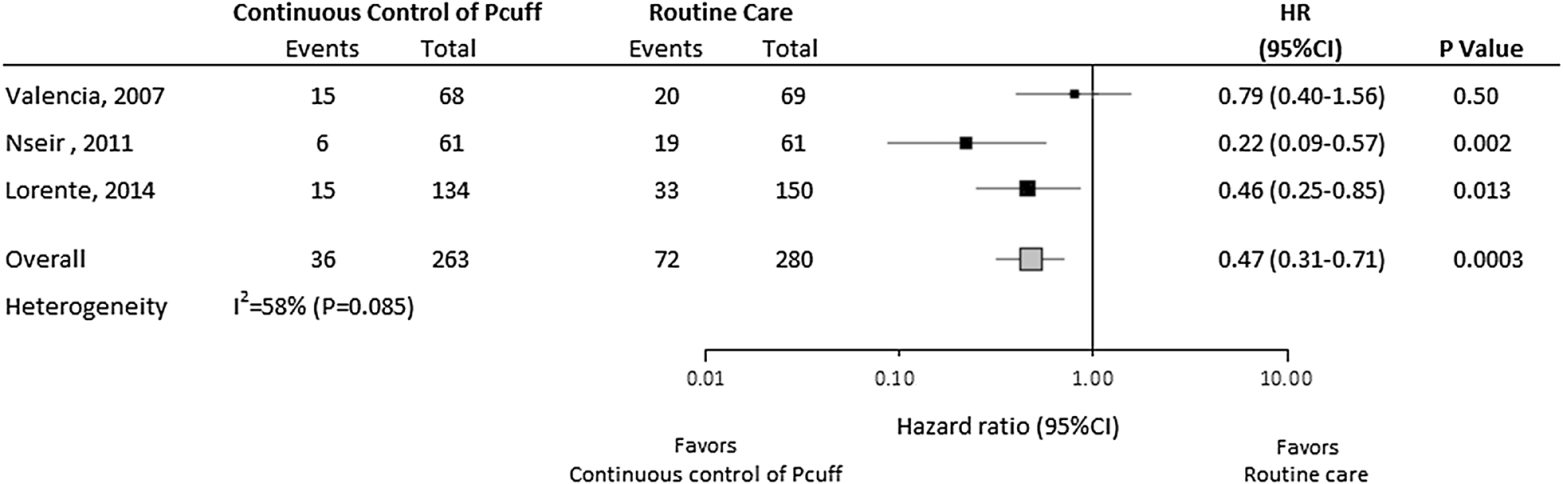
VAP-free survival curves for patients assigned to continuous control of cuff pressure and routine care groups. *p* value, and hazard ratio were calculated using Cox proportional hazard model stratified on trial

Continuous control of *P*_cuff_ was associated with significantly reduced incidence of VAP, with a HR of 0.47 (95 % CI 0.31–0.71) (Fig. 2). However, heterogeneity in continuous control effect size across trial was apparent (*I*^2^ = 58 %, *p* = 0.085). The effect of continuous control of *P*_cuff_ to reduce the incidence of VAP remained significant in exclusion sensitivity analysis, with a lower effect after exclusion of the study of Nseir and colleagues (HR 0.58; 95 % CI 0.37–0.92, *p* = 0.019). In further sensitivity analysis, excluding patients receiving subglottic secretion drainage, the effect of continuous control of *P*_cuff_ on VAP occurrence was not modified with a HR of 0.52 (95 % CI 0.33–0.79).

In multivariate cox regression analysis, continuous control of *P*_cuff_ remained significantly associated with decreased risk for VAP [HR 0.42, 95 % CI (0.27–0.64), *p* < 0.0001], independently of subglottic secretion drainage, chronic renal failure, respiratory, and digestive failures at ICU admission.

No significant difference was found in VAT rate between patients with continuous control of *P*_cuff_ and patients with routine care [13 of 263 (5 %) patients vs 22 of 280 (8 %), *p* = 0.227]. No significant impact of this procedure was found on duration of mechanical ventilation, mechanical ventilation-free days, antimicrobial treatment, length of ICU stay, or ICU mortality (Table 5).

**Table 5:** Impact of continuous control of cuff pressure on secondary outcomes

	Continuous control of *P*_cuff_		
	Yes (*n* = 263)	No (*n* = 280)	*p* value
MV duration (day)	8 (4, 16)	8 (4, 16)	0.681
MV free days	3 (0, 6)	2 (0, 5)	0.426
ICU length of stay (day)	11 (6, 24)	12 (7, 21)	0.440
Duration of antibiotic treatment	9 (6, 15)	10 (6, 15)	0.778
ICU mortality	86 (33)	91 (32)	>0.999

Data are number (%), or median (interquartile range)

*MV* mechanical ventilation, *VAP* ventilator-associated pneumonia, *ICU* intensive care unit

### Safety data and cost-effectiveness

Similar rates of tracheostomy and reintubation were found in patients who received in patients with continuous control of *P*_cuff_ compared with patients with routine care (Table 3). No significant difference was found in tracheal ischemic lesions between the two groups (*n* = 96).

The number of patients needed to treat to prevent one VAP episode was 8.

## Discussion

Our results suggest that continuous control of *P*_cuff_ might be beneficial in reducing the incidence of VAP. However, continuous control of *P*_cuff_ had no significant impact on secondary outcomes such as duration of mechanical ventilation, mechanical ventilation-free days, antimicrobial treatment, ICU stay, or ICU mortality.

### Strengths and limitations

The major strengths of this study are the large number of included patients (*n* = 543) and the collaborative-pooled design. The similarity of the trials, including intervention, disease, and study population, provided a strong rationale to pool individual patient-level data. However, some limitations of our study should be acknowledged. First, the number of included studies was small, one study was quasi-randomized, and different devices were used to continuously control *P*_cuff_ in these trials, which could probably explain the observed heterogeneity in estimates. Nevertheless, percentage of measurements of *P*_cuff_ < 20 cmH_2_O in continuous control of *P*_cuff_ group was quite similar in the three studies. Other differences between the three studies, including the lower rate of VAP in control group, and the higher percentage of patients with chronic respiratory disorders in one study [21] compared with the two others, could also explain the high heterogeneity. Second, no significant impact of continuous control of *P*_cuff_ was found on secondary outcomes, such as duration of mechanical ventilation, mechanical ventilation-free days, antimicrobial treatment, or ICU mortality. This could be explained by the fact that a larger study sample is required to demonstrate such an effect. A posteriori calculation, based on the results of the current meta-analysis, of number of patients required to demonstrate a significant impact of continuous control of *P*_cuff_ on outcomes indicates that 348 patients are required to demonstrate a reduction of VAP incidence of 12 % (from 26 to 14 %, *p* = 0.05, power 80 %), and 1132 patients are required to demonstrate a reduction of mechanical ventilation duration of 2 days (mean 14 vs 12 days, standard deviation 12, *p* = 0.05, power 80 %). Previous well-conducted randomized controlled studies aiming at evaluating a preventive measure of VAP also suffered from this limitation [14, 26, 27]. For example, several randomized controlled studies comparing subglottic secretion drainage to routine care demonstrated a significant reduction in VAP rate, but all failed to show any significant reduction in mortality rate or duration of mechanical ventilation. However, a large meta-analysis [15] performed on 2442 patients showed a significant reduction in mechanical ventilation duration in patients with subglottic secretion drainage compared to those with routine care. Third, subglottic secretion drainage was used in some patients of one trial, and might have influenced the results. However, the rate of patients who received subglottic secretion drainage was similar in patients who received a continuous control of *P*_cuff_ and those who received routine care. In addition, sensitivity analysis excluding patients who received subglottic secretion drainage did not modify the protective effect of continuous control of *P*_cuff_ on VAP incidence. Fourth, cost-effectiveness analysis could not be performed. However, the number of patients needed to treat to prevent one VAP episode was 8. Fifth, the safety of continuous control of *P*_cuff_, regarding tracheal ischemic lesions, was evaluated in only one study. Sixth, the under-reporting of negative results could have biased our results. However, we have checked the abstract of the major international critical care congresses and did not find any additional study on the impact of continuous control of *P*_cuff_ on VAP incidence.

### Explanations for study results

Rello et al. [17] have previously reported that underinflation of *P*_cuff_ was independently associated with VAP in a subgroup of patients without antimicrobial treatment. In addition, one of the three studies included in this analysis [22] has investigated the impact of continuous control of *P*_cuff_ on microaspiration of gastric content and tracheobronchial colonization. This study reported a significant (27 %) reduction of microaspiration of gastric contents, defined as the presence of pepsin in >65 % of tracheal aspirates, in patients who received continuous control of *P*_cuff_, compared with those who received routine care. Further, a significant reduction in bacterial concentration in tracheal aspirates was also observed.

Several studies reported that routine care using a manometer was not efficient in continuously controlling *P*_cuff_, and that risk factors for underinflation and overinflation of tracheal cuff were not modifiable [19, 28, 29]. Other studies clearly showed the efficiency of some devices in continuously controlling *P*_cuff_, suggesting that their use might be beneficial in preventing intubation-related complications [30, 31].

Two randomized studies, excluded from this analysis, reported a beneficial effect of continuous control of *P*_cuff_ on VAP incidence [32, 33]. However, in these studies continuous control of *P*_cuff_ was not the only tested preventive measure. While, low-volume low-pressure cuff was used in intervention group, PVC-standard cuff was used in routine care group. Therefore, it is impossible to determine whether the reduced incidence of VAP is related to continuous control of *P*_cuff_ or to low-volume low-pressure cuff use. This is the reason why these studies were not included in our analysis. Similarly, two other studies showed a beneficial effect of implementing a bundle for VAP prevention, including routine care for tracheal cuff [34, 35]. However, whether this beneficial effect is related to *P*_cuff_ control is unknown.

### Different devices for continuous control of *P*_cuff_

The heterogeneity between the three trials was high, probably reflecting the use of different devices for *P*_cuff_ control. An experimental study reported that electronic *P*_cuff_ controllers with rapid pressure correction interfere with the self-sealing mechanism of high-volume, low-pressure PVC–cuffed tracheal tubes and reduce their sealing characteristics [36]. Further, a recent prospective crossover study compared the efficiency of a pneumatic with an electronic device aiming at continuously controlling *P*_cuff_ [37]. The authors found underinflation of tracheal cuff to be more frequent using the electronic device than the pneumatic device (7 vs 0 %, respectively) and attributed this result to the over compensation of any elevated *P*_cuff_.

### Future studies

Future randomized controlled multicenter studies are required to confirm the beneficial effect of continuous control of *P*_cuff_ on VAP incidence, before recommending its routine use. In addition, the efficiency of different available devices should also be compared in critically ill patients.

## Conclusions

Continuous control of *P*_cuff_ might be beneficial in reducing the incidence of VAP. However, no significant impact of this preventive measure was found on duration of mechanical ventilation, mechanical ventilation-free days, antimicrobial treatment, or ICU mortality. Further studies are required to confirm these results and to evaluate safety and cost-effectiveness of this preventive measure of VAP.

## Abbreviations

APACHE: Acute physiology and chronic health evaluation
COPD: Chronic obstructive pulmonary disease
ICU: Intensive care unit
*P*_cuff_: Cuff pressure
PEEP: Positive end expiratory pressure
PVC: Polyvinyl chloride
SOFA: Sequential organ dysfunction assessment
VAP: Ventilator-associated pneumonia
